# Synthesis of Gd-DTPA Carborane-Containing Compound and Its Immobilization on Iron Oxide Nanoparticles for Potential Application in Neutron Capture Therapy

**DOI:** 10.3390/pharmaceutics16060797

**Published:** 2024-06-12

**Authors:** Ilya V. Korolkov, Alexander Zaboronok, Kairat A. Izbasar, Zhangali A. Bekbol, Lana I. Lissovskaya, Alexandr V. Zibert, Rafael I. Shakirzyanov, Luiza N. Korganbayeva, Haolan Yang, Eiichi Ishikawa, Maxim V. Zdorovets

**Affiliations:** 1 The Institute of Nuclear Physics, Ibragimov Str. 1, 050032 Almaty, Kazakhstan; kaira.kim31@gmail.com (K.A.I.); zhangali.bekbol@mail.ru (Z.A.B.); ms.defrance@mail.ru (L.I.L.); mzdorovets@inp.kz (M.V.Z.); 2 Engineering Profile Laboratory, L.N. Gumilyov Eurasian National University, Satpaev Str. 5, 010008 Astana, Kazakhstan; shakirzyanov_ri@enu.kz (R.I.S.); luiza.korganbaeva@yandex.ru (L.N.K.); 3 Department of Neurosurgery, Institute of Medicine, University of Tsukuba, 1-1-1 Tennodai, Tsukuba 305-8575, Japan; s2430438@u.tsukuba.ac.jp (H.Y.); e-ishikawa@md.tsukuba.ac.jp (E.I.)

**Keywords:** neutron capture therapy, nanoparticles, iron oxide, carborane, Gd-DTPA, drug delivery

## Abstract

Cancer is one of the leading causes of global mortality, and its incidence is increasing annually. Neutron capture therapy (NCT) is a unique anticancer modality capable of selectively eliminating tumor cells within normal tissues. The development of accelerator-based, clinically mountable neutron sources has stimulated a worldwide search for new, more effective compounds for NCT. We synthesized magnetic iron oxide nanoparticles (NPs) that concurrently incorporate boron and gadolinium, potentially enhancing the effectiveness of NCT. These magnetic nanoparticles underwent sequential modifications through silane polycondensation and allylamine graft polymerization, enabling the creation of functional amino groups on their surface. Characterization was performed using Fourier-transform infrared spectroscopy (FTIR), X-ray diffraction (XRD), energy dispersive X-ray (EDX), dynamic light scattering (DLS), thermal gravimetric analysis (TGA), and transmission electron microscopy (TEM). ICP-AES measurements indicated that boron (B) content in the NPs reached 3.56 ppm/mg, while gadolinium (Gd) averaged 0.26 ppm/mg. Gadolinium desorption was observed within 4 h, with a peak rate of 61.74%. The biocompatibility of the NPs was confirmed through their relatively low cytotoxicity and sufficient cellular tolerability. Using NPs at non-toxic concentrations, we obtained B accumulation of up to 5.724 × 10^10^ atoms per cell, sufficient for successful NCT. Although limited by its content in the NP composition, the Gd amount may also contribute to NCT along with its diagnostic properties. Further development of the NPs is ongoing, focusing on increasing the boron and gadolinium content and creating active tumor targeting.

## 1. Introduction

Cancer is the second leading cause of death worldwide, primarily due to its aggressive nature and the challenges associated with developing effective treatment strategies. According to the latest estimates, the global incidence of cancer reached 19,976,499 cases, and the mortality rate was 9,743,832 people in 2022 [1]. Cancer is typically treated through a combination of surgery, chemotherapy, and radiation therapy. These methods collectively aim to eliminate or control the growth of cancer cells and improve the chances of successful treatment.

Radiotherapy is an essential and widely used method of cancer treatment. Although its efficacy has been extensively demonstrated, certain types of tumors, such as adenocarcinomas, sarcomas, carcinomas, and gliomas, are considered radioresistant, exhibiting little or no response to radiotherapy [2]. This resistance is partly attributed to the use of X-ray radiation therapy, which has low linear energy transfer (LET). While proton therapy may offer increased effectiveness in some cases, it comes with its own set of limitations and is unable to completely cure certain high-malignancy tumors, including gliomas. Neutron capture therapy (NCT) emerges as a promising alternative that is capable of overcoming the limitations of other radiotherapy methods. It is a binary technology that utilizes neutron radiation along with compounds based on elements capable of capturing neutrons and locally releasing large amounts of energy [3,4,5]. NCT with a ^10^B compound (boron neutron capture therapy, or BNCT) can deliver high-LET radiation to radioresistant hypoxic cancer cells in rapidly growing tumors [6,7]. With the tumor-targeting properties of compounds designed for NCT, this technology can target individual cancer cells during their invasion while minimally impacting healthy tissues. The development and introduction of accelerator-based neutron sources suitable for installation in medical facilities led to the transition of BNCT from nuclear reactors to these accelerator systems and its clinical approval in Japan in 2020 [6,7,8,9,10,11,12,13,14,15]. This approval included government insurance coverage for treating specific tumors, marking a significant transition of NCT from clinical trials to clinical application. This progression has stimulated global research into developing new, more advanced compounds for this technique.

Various agents have been proposed for neutron capture therapy (NCT), such as ^10^B and ^157^Gd. Treatments involving these specific agents are known as BNCT (boron neutron capture therapy) and GdNCT (gadolinium neutron capture therapy). NCT employs a technique that utilizes agents with a high affinity for neutron capture, often enriched with isotopes like boron-10 (^10^B) or gadolinium-157 (^157^Gd), selectively delivered to cancer cells. Upon exposure to a thermal neutron flux, these isotopes undergo rapid decomposition through the neutron capture reaction, primarily emitting α-particles. Both the recoiling nucleus and α particle have a confined travel range, approximately equivalent to the dimensions of a mammalian cell. Additionally, they exhibit a high linear energy transfer, signifying the deposition of substantial ionizing energy within this limited range. This process leads to DNA damage, thereby initiating the apoptosis process within the cancer cells [16,17,18,19].

The effective delivery of NCT agents to target cells within a tumor is a crucial aspect of cancer treatment via neutron capture therapy (NCT). This process necessitates low toxicity, optimal accumulation, and sustained presence in the tumor during neutron irradiation, along with rapid clearance from normal tissues and blood. Meeting these requirements is essential for the success of NCT [20]. Nanomaterials emerge as promising candidates for facilitating the targeted delivery of isotopes to different cancers. Their multifunctionality makes them ideal targeting vectors, allowing for conjugation with various cancer-specific ligands. This versatility enhances the selective delivery of an adequate amount of NCT agents to tumor cells, addressing the key prerequisites for effective cancer treatment via NCT [21,22,23].

In this context, the delivery of agents can be facilitated through the immobilization of magnetic NPs and the manipulation of an external magnetic field. This approach enables precise control over the transport and localization of the agents within the targeted areas, offering a promising strategy for enhancing the efficiency and specificity of drug delivery systems. The immobilization of magnetic NPs allows for increased stability and targeted release, ensuring optimal therapeutic outcomes [24].

Sodium mercaptoundecahydro-closo-dodecaborate (BSH) and (L)-4-dihydroxy-borylphenylalanine (L-BPA) are two boron-containing agents that have received clinical approval. These compounds play a crucial role in boron neutron capture therapy (BNCT). BSH, first synthesized over 50 years ago, was initially employed by Hiroshi Hatanaka in the 1960s for BNCT of high-grade gliomas. On the other hand, L-BPA, synthesized around the same time, saw its clinical debut in 1988 when Mishima used it to treat patients with cutaneous malignant melanomas [25,26,27,28].

L-BPA has emerged as the most widely used boron compound in BNCT clinical trials. Typically delivered as a water-soluble fructose or sorbitol complex, it is enriched with ^10^B and labeled with ^18^F for positron emission tomography (PET) imaging before neutron irradiation. The BPA-fructose complex for BNCT of patients gained prominence in 1994, specifically for treating glioblastoma. Since then, BPA-fructose has become the preferred clinical boron delivery agent for both intra- and extra-cranial tumors [29,30,31].

Low molecular weight agents form a cornerstone in BNCT, presenting a promising pathway for targeted cancer treatment. Amino acids such as aspartic acid, methionine, glycine, cysteine, alanine, and tyrosine and their non-naturally occurring counterparts have been strategically employed in BNCT. This delivery system excels in efficacy, ensuring the precise conveyance of high boron concentrations to tumors while mitigating adverse effects [16,20,32,33].

The success of low molecular weight agents is further propelled by polyhedral borane dianions like closo-B_10_H_10_^2−^ and closo-B_12_H_12_^2−^, as well as icosahedral carboranes like closo-C_2_B_10_H_12_ and nido-C_2_B_9_H_12_^−^. These structures, characterized by their hydrophobic nature, negative charge, chemical stability, and elevated boron content, collectively enhance their suitability for BNCT applications [16].

In therapeutic interventions, high molecular weight delivery agents assume a pivotal role, with monoclonal antibodies (mAbs) and nanomaterials emerging as prominent contributors. Studies investigate the efficacy of mAb cetuximab and epidermal growth factor (EGF) as delivery agents targeting the mutant isoform of the EGF receptor, prevalent in tumors like squamous cell carcinomas of the head and neck and gliomas [20,34].

Various delivery approaches encompass both active and passive targeting strategies. Passive targeting leverages the enhanced permeability and retention (EPR) effect, capitalizing on NPs’ ability to navigate vascular architecture. Conversely, active targeting involves attaching specific antibodies or ligands to molecules or nanocarriers, directing them toward tumor cells for a more precise therapeutic impact. This nuanced interplay of low and high molecular weight agents underscores the multifaceted strategies employed in advancing cancer therapies [20].

However, some researchers state that there are advantages to utilizing both boron and gadolinium compounds in neutron capture technology [18,24,35]. This approach highlights the additional diagnostic properties and synergistic effects observed in neutron capture reactions. The immobilization of Gd ions can be achieved using chelate complexes, like Gd-DTPA [36,37]. This author showed us that the inclusion of Gd-DTPA has been reported to significantly reduce the survival rate of cancer cells by 80–90%.

In our previous research [38], we showed the simultaneous immobilization of a carborane compound and Gd ions on Fe_3_O_4_ NPs; however, it was found that there was a rapid release of gadolinium ions.

In the present study, Gd-DTPA, a stable complex with medical applications, was combined with a carborane derivative of hydrindon. Subsequently, agents containing both boron and gadolinium were immobilized onto modified magnetic NPs, demonstrating a novel approach to enhance the stability and functionality of magnetic nanomaterials for their potential medical applications.

## 2. Materials and Methods

### 2.1. Chemicals

Iron(II) chloride tetrahydrate, iron(III) chloride hexahydrate, 3-(Trimethoxysilyl)propyl methacrylate (TMSPM) (98%), allylamine (98%), trimethylolpropane trimethacrylate (TMPTMA), benzoyl peroxide (75%), aluminum oxide, dietylenetriaminepentaacetic acid gadolinium(III) dihydrogen salt hydrate (GD-DTPA) (97%), N,N-dicyclohexylcarbodiimide (DCC) (99%), pentafluorophenol (PFP) (99%), sodium hydroxide, sodium borohydride, iron(III) nitrate nonahydrate, copper(II) acetate, acetonitrile, n-propanol, isopropyl alcohol, n-butyllithium, and tetrahydrofuran (THF) were purchased from Sigma-Aldrich (Merck KGaA, Darmstadt, Germany). Sodium chloride, ethanol, o-xylene, diethyl ether, dimethylformamide (DMF), chloroform, benzene, hydrochloric acid (HCl), isopropyl-o-carborane, hexane, nitro-malonic ether, ethyl acetate, sodium sulfate, acetic acid, and hydrobromic acid were of chemical grade. In all experiments, deionized water (18.2 MΩ·cm) was used.

### 2.2. Synthesis of Fe_3_O_4_ NPs and Their Modification

To a solution of 100 mL deionized water and 11.8 mL HCl, 9.94 g of FeCl_2_·4H_2_O and 27.029 g of FeCl_3_·6H_2_O were added. The reaction was conducted under a flow of argon at 80 °C. After two hours, the black precipitate was separated using a magnet, then washed several times with water and ethanol, and dried in a Petri dish at 60 °C in air.

### 2.3. Functionalization of Fe_3_O_4_ NPs

#### 2.3.1. Double-Bond Formation on NPs

To an ultrasonic bath, 100 mL o-xylene and 1 g Fe_3_O_4_ NPs were added for 2 h. Subsequently, 0.0126 mol TMSPM was added. The reaction proceeded under mechanical stirring for 5 h at a temperature of 80 °C. The precipitate was then magnetically separated, washed three times with o-xylene, three times with diethyl ether, and dried in air.

#### 2.3.2. Graft Polymerization of Allylamine and Cross-Linking

Allylamine and TMPTMA were preliminarily purified by filtration through a column of aluminum oxide. Next, 1 g of NPs was added to 79.4 mL of dimethylformamide (DMF). The solution was maintained under a flow of argon. Then, 10.38 mL of allylamine and 0.638 mL of TMPTMA (cross-linking agent), as well as 0.048 g of benzoyl peroxide (initiator), were added. Previously, the monomers had been purified of the inhibitor using an aluminum oxide column. The temperature was maintained at 80 °C for 24 h, and the reaction was mechanically stirred. Subsequently, the product was washed several times with dimethylformamide and isopropyl alcohol.

### 2.4. Synthesis of Gd-DTPA-GDD

#### 2.4.1. Synthesis of Modified Gd-DTPA

**Figure 1:**
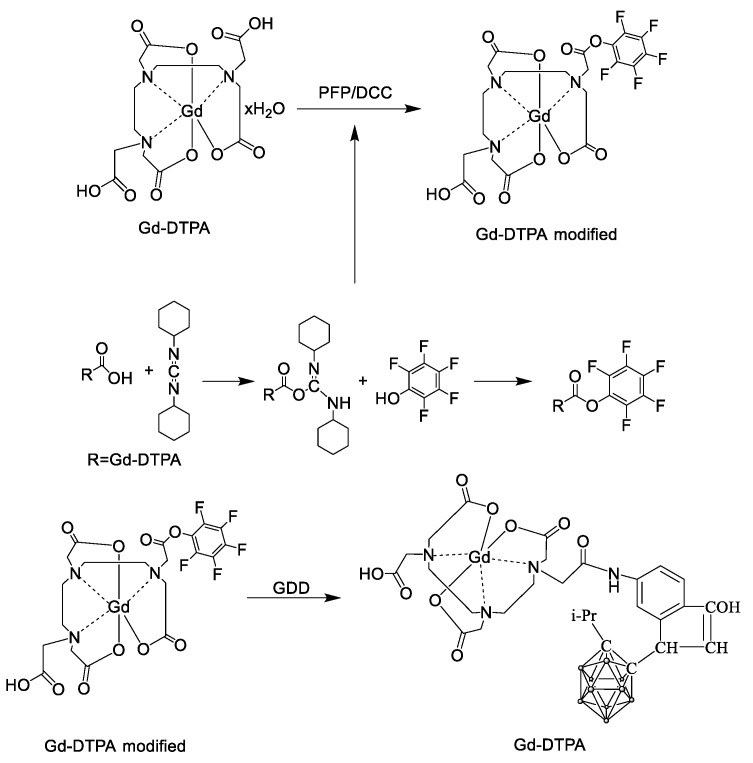
Scheme of DTPA modification and reaction with GDD.

A 100 mL solution was prepared from deionized water and acetonitrile in a 2:3 ratio, and 1 g of GD-DTPA was added to water adjusted to pH 10 (using NaOH). Then, 0.336 g of PFP and half of the solution were added. Subsequently, 0.378 g of DCC was dissolved separately in a solution of deionized water and acetonitrile (2:3 ratio). Both mixtures were left to stir overnight at room temperature. Afterward, the solution was evaporated. The product was recrystallized in acetonitrile and water (3:1 ratio). The scheme of Gd-DTPA modification is presented in Figure 1.

The FTIR spectrum of the product shows main peaks at 3300 cm^−1^ (-OH), 2900 cm^−1^ (CH_2_), 1600 cm^−1^ (C=O), 1400 cm^−1^ (C-F), 1493, 1440, 1325, 1270, 1239, 1217, 1168, 1122, 1093, 1058, 1034, 1007, 979, 930, 897, 868, 848, 804, 711, and 448 cm^−1^.

#### 2.4.2. Synthesis and Reduction of 2,3-(3-Nitrophenyl)-4-(isopropyl-o-carboranyl)hydrindone (GDD-NO_2_)

**Figure 2:**
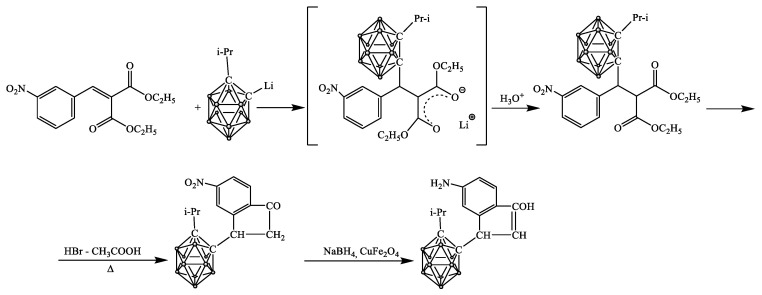
Scheme of synthesis and reduction of 2,3-(3-nitrophenyl)-4-(isopropyl-o-carboranyl)hydrindone (GDD-NO_2_).

The synthesis and reduction of 2,3-(3-nitrophenyl)-4-(isopropyl-o-carboranyl)hydrindone (GDD-NO_2_) were carried out according to the scheme presented in Figure 2.

At the first stage, in a 250 mL three-neck flask equipped with a stirrer and under argon, 7.65 mL (0.044 mol) isopropyl-o-carborane, 20 mL of hexane, and 18.3 mL (2.4 N) solution of n-butyllithium in hexane was added. The reaction mixture was stirred for about half an hour until a white precipitate formed. Next, the precipitate was dissolved by adding 30 mL of tetrahydrofuran. The flask with the reaction mixture was cooled to a temperature of −50 °C using a cryostat. After that, 15 mL (0.044 mol) of nitro-malonic ether was added. The reaction mixture was stirred for about 5–6 h while increasing the temperature by 10 °C every hour. After reaching a temperature of 0 °C, the reaction mixture was also stirred for about an hour, then the cryostat was turned off, and the reaction mixture was stirred at room temperature. The reaction completion was monitored by thin-layer chromatography (TLC). After the reaction was completed, the mixture was treated with hydrochloric acid until neutral, extracted with ethyl acetate, and left to dry under sodium sulfate for a day. Next, the substance was filtered from sodium sulfate through a funnel, and the remaining solvent was evaporated on a rotary unit, and a resinous product was obtained. This product was crystallized with hexane and then recrystallized.

Then, 4 g (0.0083 mol) of isopropyl-*o*-carboranyl-*m*-nitrobenzylidenemalonate, 42.5 mL of glacial acetic acid, and 15.31 mL of hydrobromic acid were added to a 250 mL round-bottom flask equipped with a magnetic stirrer and reflux condenser. The mixture was heated to boiling overnight. At the end of the reaction, the mixture was cooled to room temperature. The resulting precipitate was filtered on a Schott filter and washed repeatedly with water. Next, the precipitate was dried and recrystallized in benzene. The product yield was 89%, m.p. 192–195 °C.

The FTIR spectrum contains absorption bands in the regions: 2580 and 2630 cm^−1^ valence vibrations of B-H belonging to the carborane core. The peak at 1710 cm^−1^ is the (C=O) group, and 1300 cm^−1^ is plane deformation ring vibrations of the benzene ring. The peak at 930 cm^−1^ corresponds to out-of-plane bending vibrations of the benzene ring. The peak at 1390 cm^−1^ is NO_2_. Peaks at 1432 cm^−1^ and 1479 cm^−1^ are valence vibrations of the benzene ring.

NMR H^1^ (in DMSO) ppm (J, Hz): 8.24 (s., 1H, C_sp2_H), 8,21 (d., J = 1.4 Hz, 1H, C_sp2_H), 7.92 (d., J = 7.7 Hz, 1H, C_sp2_H), 4.11 (m., 1H, C-H), 2.87 (m., 2H, CH_2_), 2.39–2.34 (m, 1H, CH(CH_3_)_2_), 1.24 (dd., J = 6.7, 2.5 Hz, 6H, CH(CH_3_)_2_), 1.30–2.40 (10H, m., BH).

The catalyst for NO_2_-GDD reduction was synthesized according to the procedure described in [39]. For this purpose, Cu(CH_3_COO)_2_·H_2_O, Fe(NO_3_)_3_·9H_2_O, NaOH, and NaCl were mixed in a ratio of 1:2:8:2 and ground in an agate mortar for 50 min. The reaction was accompanied by the release of heat. The mixture gradually changed color from blue to brown. The mixture was then washed several times with deionized water.

Then, the reduction of compound 1 (NO_2_-GDD) was carried out according to the following procedure: 1 g of NO_2_-GDD was dissolved in n-propanol until completely dissolved, followed by the addition of 1 g of CuFe_2_O_4_ (catalyst) [40,41] and 9.51 mL of H_2_O. Next, 5 g of NaBH_4_ was added to the solution. The reaction was conducted at room temperature with a magnetic stirrer for 12 h.

The primary objective of the synthesis process is to effectively capture hydrogen (H_2_), achieved through the chemical reaction between sodium borohydride (NaBH_4_) and water (H_2_O). This reaction facilitates the generation and subsequent utilization of H_2_ for the reduction of nitro compounds, utilizing a copper iron oxide (CuFe_2_O_4_) catalyst. The resulting hydrogen (H_2_) is then collected in a containment vessel, typically a balloon, for use during this chemical reaction.

After the reaction, the resultant yellow liquid was separated from the mixture and dried. Subsequently, the obtained powder underwent recrystallization processes facilitated by benzene and chloroform solvents.

The reduction of NO_2_-GDD resulted in the emergence of peaks in the FTIR spectra at 3300–3000 cm^−1^ (O-H, N-H) and 1413 cm^−1^ (C-O), replacing the C=O bond observed at 1700 cm^−1^. Restoration of the N-O bond was evidenced by peaks at 1500 cm^−1^ and 1360 cm^−1^, leading to the appearance of an N-H peak at 1653 cm^−1^. Additionally, the B-H peak remained prominent at around 2600 cm^−1^. Other observed peaks include 2979, 2940, and 2880 cm^−1^ (C-H stretching), as well as peaks at 1576, 1461, 1397, 1372, 1310, 1189, 1171, 1127, 1083, 1015, 999, 924, 907, 874, 792, 734, 700, and 664 cm^−1^.

#### 2.4.3. Synthesis of Gd-DTPA-GDD

GDD (0.25 g) was dissolved in 80 mL of isopropyl alcohol, while 0.53 g of modified Gd-DTPA was dissolved in 20 mL of water. The reaction mixture was stirred for 24 h at a temperature of 50 °C. TLC was used to monitor the reaction completion. Subsequently, the solution was evaporated, and recrystallization was carried out using a mixture of ethyl acetate and hexane in a 2:3 ratio. FTIR: 3200 cm^−1^ (O-H), 2600 cm^−1^ (B-H), 1660 cm^−1^ (CO-NH), 1580 cm^−1^, 1500 cm^−1^, 1430 cm^−1^, 1300 cm^−1^, 1241, 1175, 1012, 979, 881, 805, 734, 700, and 653.

### 2.5. Immobilization of Gd-DTPA-GDD in Functionalized Fe_3_O_4_ NPs

Functionalized NPs (0.1 g) and 0.0117 g of Gd-DTPA-GDD were added to 10 mL of deionized water. The pH was adjusted accordingly. Analysis revealed that the highest concentration was achieved at pH 8 (using NaOH). The reaction mixture was then placed on a shaker for 24 h at room temperature.

### 2.6. Gd-DTPA-GDD Release from Nanoparticles

**Figure 3:**
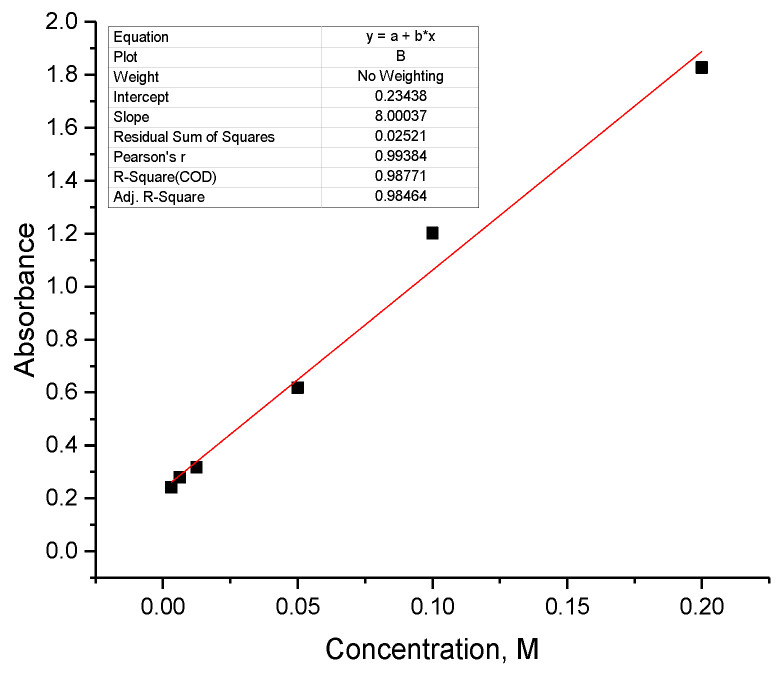
Calibration curve of DTPA-GDD.

One PBS tablet (Sigma-Aldrich, Merck KGaA, Darmstadt, Germany) was added to 100 mL of deionized water and dissolved. In 14 mL of this solution, 0.07 g of Fe_3_O_4_-TMSPM-PAlAm/Gd-DTPA-GDD was added. The sample remained on the shaker at 36.6 °C. The sample was taken out after 5, 10, 15, and 30 min, then after 1, 2, and 4 h. After this, the NPs were analyzed every 24 h for a week. The final analysis transpired 126 h post-initiation. The concentration of DTPA-GDD was evaluated by UV-vis spectroscopy (at 273 nm) using a calibration plot (Figure 3, R^2^ = 0.98464).

### 2.7. Methods of Characterization

FTIR spectra were recorded using an InfraLUM FT-08 FTIR spectrometer (Lumex Instruments, St. Petersburg, Russia), operating in the range of 400–4000 cm^−1^ with an ATR accessory. Measurements were conducted over 25 scans at a resolution of 2 cm^−1^.

SEM-EDX analysis was conducted using a Hitachi TM 3030 scanning electron microscope (Hitachi High-Technologies Corporation, Tokyo, Japan) equipped with a Bruker XFlash MIN SVE microanalysis system (Bruker Corporation, Billerica, MA, USA), operating at 15 kV.

X-ray diffraction analysis was conducted using a D8 ADVANCE ECO diffractometer (Bruker, Karlsruhe, Germany) equipped with a CuKα radiation source (λ = 1.54060 Å). For phase identification and crystal structure analysis, Bruker AXS DIFFRAC.EVA software version 4.2 and the International Centre for Diffraction Data (ICDD) PDF-2 database were used.

The size of NPs was analyzed using a NanoBrook 90Plus Zeta particle size analyzer (Brookhaven Instruments, Holtsville, NY, USA) in PBS solution at 36.6 °C with 5 min stabilization time. Zeta potential was measured at 25 °C in ELS mode (phase analysis light scattering) using Smoluchowski’s approximations. The pH of the PBS solution was adjusted using phosphoric acid and sodium hydroxide.

Additionally, particle size and shape were studied using a JEM-1400 transmission electron microscope operated at 80 keV (TEM, JEOL, Tokyo, Japan).

The surface area, pore volume, and pore width were determined by BET analysis using He/N_2_ gases on a V-Sorb 2800P surface area analyzer (Gold APP Instruments, Corp., Ltd., Hong Kong, China). Before measurements, samples were pretreated by a degassing procedure at 120 °C.

Pyris 1 TGA (Perkin-Elmer, Waltham, MA, USA) was used for thermogravimetric analysis in the temperature range of 30 to 700 °C with a programmed temperature increment of 10 °C min^−1^ in a nitrogen atmosphere.

**Figure 4:**
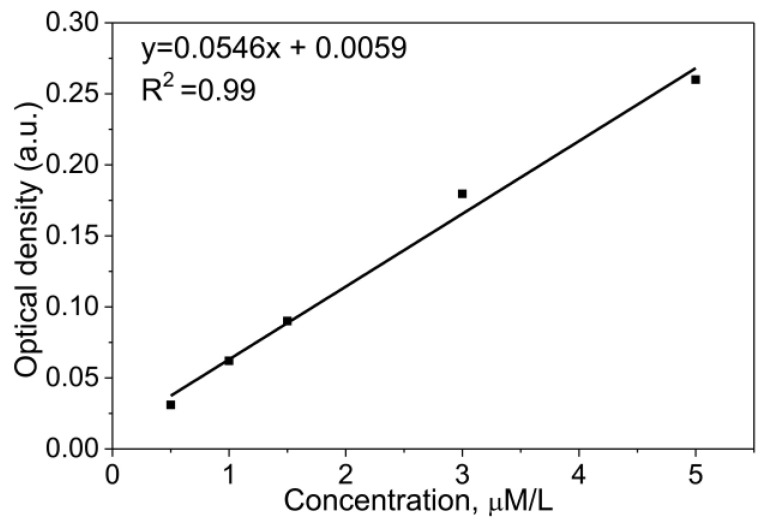
Calibration curve of acid orange.

Amino groups were determined using acid orange (AO) dye according to the procedure described in [42]. In brief, NPs (0.085 g) were immersed in a 500 μmol/L AO solution (in HCl, pH = 3) for 12 h to allow the binding of the AO to the nanoparticle surface. Subsequently, nanoparticles were separated from the solution using a magnet and decanted. Desorption of each sample was conducted in 5 mL of NaOH solution (pH = 12) for 15 min on a shaker. Amino group concentration was quantified via spectrophotometric measurements at 495 nm using a calibration plot (Figure 4, R^2^ = 0.99).

### 2.8. Stock Solution Preparation and Elemental Analysis

Fe, B, and Gd content in the particles and their stock solutions was evaluated using inductively coupled plasma atomic emission spectroscopy (ICP-AES; ICPS-8100 Twin Sequential ICP Emission Spectrometer, Shimadzu, Inc., Tokyo, Japan). For this, particle powder was weighed, and seven samples with average weights ranging from 0.01 to 0.1 g were diluted in 1 to 5 mL of phosphate-buffered saline (PBS). The solution was sonicated at 20 kHz using a 130 W Sonics VibraCell™ VCX 130PB ultrasonic processor (Sonics & Materials, Inc., Newton, CT, USA) until a homogeneous suspension of NPs was achieved. Between experiments, prepared stock solutions were stored at −20 °C to prevent Gd release from the NPs. For elemental analysis, samples of 10, 50, and 100 µL from the stock solutions were placed in 1 mL of concentrated HNO_3_. Next, 3 mL of concentrated HCl was added to form Aqua Regia, and the solutions were boiled at 115 °C for 1 h in heat-resistant polypropylene tubes. Then, Milli-Q water was added to adjust the final volume to 5 or 10 mL, and the samples were filtered through 0.20 µm filters and analyzed using ICP-AES. The most suitable wavelengths selected were 259.940 nm for Fe, 249.773 nm for B, and 342.247 nm for Gd. The average of three measurements for each sample was used to characterize the element content in ppm.

### 2.9. Human Glioblastoma Cell Line

T98G cells were purchased from the American Type Culture Collection (ATCC, Manassas, VA, USA), cultured in Minimum Essential Medium Eagle with L-glutamine and sodium bicarbonate (Sigma-Aldrich, Catalog #M4655, Merck KGaA, Darmstadt, Germany) with 10% fetal bovine serum (HyClone™, Catalog #SH30396.03, GE Healthcare Life Sciences, South Logan, UT, USA) and 1% Penicillin–Streptomycin (10,000 units/mL penicillin and 10 mg/mL streptomycin (Sigma-Aldrich, Catalog #P0781, Merck KGaA, Darmstadt, Germany) and incubated at 37 °C in 5% CO_2_.

### 2.10. Cytotoxicity Assay

Nanoparticle cytotoxicity was assessed using MTS assay according to the previously described protocols [43,44]. In short, T98G cells were seeded in 96-well plates and incubated for 24 h in the amount of 4 × 10^4^ cells in 100 µL of medium per well. The medium was aspirated and replaced with the medium containing NPs in concentrations of 0–1500 µg Fe/mL and incubated for the next 24 h. The NP-containing medium was then removed, and the wells were washed with PBS. Then, 100 µL of the MTS solution with the mixture of 1 part of 3-(4,5-dimethylthiazol-2-yl)-5-(3-carboxymethoxyphenyl)-2-(4-sulfophenyl)-2H-tetrazolium with PMS (Cell Titer 96^®^ AQueous One Solution, Promega Corporation, Madison, WI, USA) and 5 parts of the medium was added to each well. The plates were further incubated for 2 h. As accumulated in cells, nanoparticles can absorb light and interfere with the results of the experiment; the MTS solution was transferred from the cell-containing plates to the clean plates before the analysis. The 490 nm light absorption was measured using a Bio-Rad Model 2550 EIA plate reader (Bio-Rad Inc., Hercules, CA, USA), and cell proliferation results were presented as ratios compared to controls without NPs.

### 2.11. Nanoparticle Accumulation in Tumor Cells

Active element accumulation was analyzed using ICP-AES based on the previously adopted protocols [43,44,45]. T98G cells were seeded in 25 cm^2^ flasks in the amount of 10^6^ cells per flask in 3 mL of medium and incubated for 24 h. NPs in the amounts of 50, 100, and 100 ppm of Fe were added to the medium, and the cells were further incubated for 24 h. After that, to remove NPs from the residual medium, the cells were washed three times with PBS, trypsinized using L-Trypsin-EDTA solution (2.5 g L-Trypsin, 1 mmol L-EDTA; Nacalai Tesque, Inc., Kyoto, Japan, Catalog #35554-64), placed in heat-resistant polypropylene tubes, counted, and centrifuged. Then, 1 mL of concentrated HNO_3_ and 3 mL of concentrated HCl were added to the sedimented cells to form Aqua Regia, and the solutions were boiled at 115 °C for 2 h. Milli-Q water was added to form the final volume of 6 mL, and the samples were filtered through 0.20 µm filters and analyzed using ICP-AES.

## 3. Results

**Figure 5:**
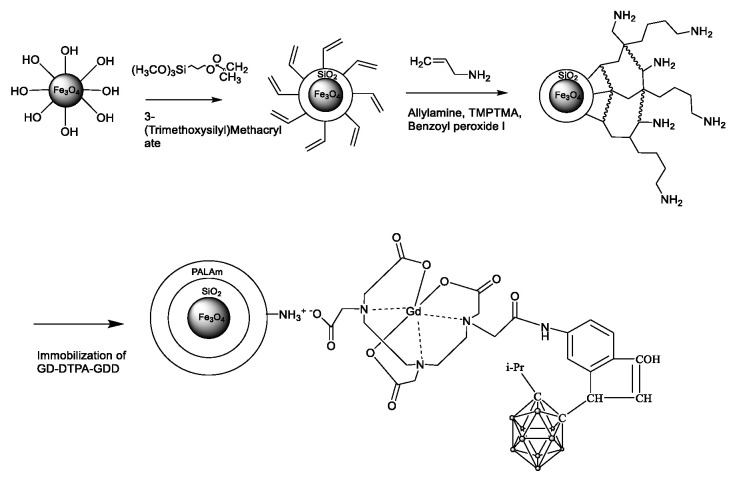
Scheme of Fe_3_O_4_ modification and Gd-DTPA-GDD immobilization.

The schematic representation of Fe_3_O_4_-NP modification is shown in Figure 5.

**Figure 6:**
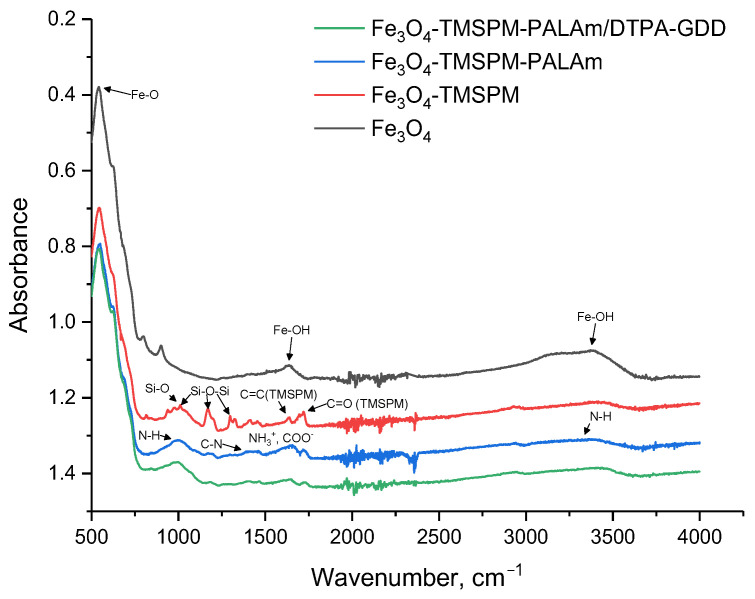
FTIR of Fe_3_O_4_ at different stages of modification and adsorption of DTPA-GDD.

In the FTIR spectrum of initial Fe_3_O_4_ NPs, three prominent bands are evident at wavenumbers of 580 cm⁻^1^, 1630 cm⁻^1^, and 3405 cm⁻^1^ [40,41], as indicated in Figure 6.

Following the procedure outlined in Figure 1, the surface of Fe_3_O_4_ underwent functionalization through the C=C double bonds via a polycondensation reaction with TMSPM. The 1170, 1012 cm^−1^, and 1635 cm^−1^ bands are described by Si-O-Si and C=C bonds. Moreover, the 1296 cm^−1^ bond can be ascribed to the stretching vibrations of Si-O-Si bonds. The 937 cm^−1^ bonds are indicative of Si-OH, suggesting an incomplete reaction process [46,47].

The next stage of modification was the graft polymerization of allylamine and its cross-linking using the TMPTMA agent. Based on the FTIR spectrum of the sample Fe_3_O_4_-TMSPM-PAlAm, NH groups are identifiable at 1650 and 3500 cm^−1^, while CH bonds were observed at 1385 cm^−1^. Following the immobilization of Gd-DTPA-GDD onto functionalized Fe_3_O_4_ NPs, the FTIR spectrometer was unable to detect any bonds at 2600 cm^−1^ (B-H). This is probably due to the fact that the concentration of DTPA-GDD is below the detection limit of the ATR-FTIR spectra. However, broad adsorption at 1600–1680 cm^−1^ was detected, which may be related to NH_3_^+^ of PAlAm and COO^−^ bonds of Gd-DTPA-GDD, which indicates their interaction.

The elemental content of NPs was evaluated by EDA. After modification of NPs with TMSPM, carbon (5.63%) and silica (0.98%) were detected. Graft polymerization of allylamine led to the detection of nitrogen (0.7%) with an increase in carbon content to 6.94%. The adsorption of Gd-DTPA-GDD led to the detection of boron (3.89%) and gadolinium (0.28%). The details are summarized in Table 1.

The content of boron and gadolinium was also elucidated by ICP-MS since the quantitative content of these elements is important for calculating the dose of the drug. According to ICP-MS, the average content of B in NPs is 3.56 ppm/mg, and that of Gd is 0.26 ppm/mg.

Additionally, the amino group concentration on the surface of Fe_3_O_4_-TMSPM-PAlAm was studied to establish optimal conditions for graft polymerization of allylamine. The results are presented in Table 2.

There is a decrease in the concentration of allylamine with increasing amounts of the substance. This is due to the fact that at high concentrations of the monomer, the formation of homopolymers occurs. Therefore, the most effective concentration of allylamine was selected at 0.18 M, which led to an amino-group concentration of 15.2 μmol/g.

Data on the surface area and pore analysis of the samples are presented in Table 3. The analysis was performed using He/N_2_. The specific surface area of the initial Fe_3_O_4_ nanoparticles is 102.161 m^2^/g, with a total pore volume of 0.34 cm^3^/g and an average pore width of 13.31 nm. After coating Fe_3_O_4_ with TMSPM, the surface area and total pore volume decrease to 64.03 m^2^/g and 0.28 cm^3^/g, respectively. The formation of a polymer layer subsequently increases both surface area and pore volume to 86.95 m^2^/g and 0.30 cm^3^/g. As expected, drug sorption leads to a decrease in pore volume (to 6.97 cm^3^/g) and surface area (to 67.15 m^2^/g) due to pore filling. The surface energy of the samples was calculated indirectly based on adsorption isotherm data using the following equations.

The surface energy was calculated using the following equations:

(1) *E_a_* = *E_L_* + *RT* ln(*C*)

where *E_a_* is the adsorption energy, *E_L_* is the liquefaction energy (cohesion energy) of the gas, *R* is the gas constant (8.314 J/(mol·K)), *T* is the temperature in Kelvin, and *C* is the BET constant, calculated from the gas adsorption isotherm, and

(2) $$\gamma =\frac{E_{a}}{S\times m}$$

where *γ* is the surface energy, *S* is the Langmuir area, and *m* is the weight of the substance.

An increase in specific surface energy is observed for PalAm-coated nanoparticles, which can be explained by the formation of a large number of polar amino groups capable of charging and forming hydrogen bonds.

**Figure 7:**
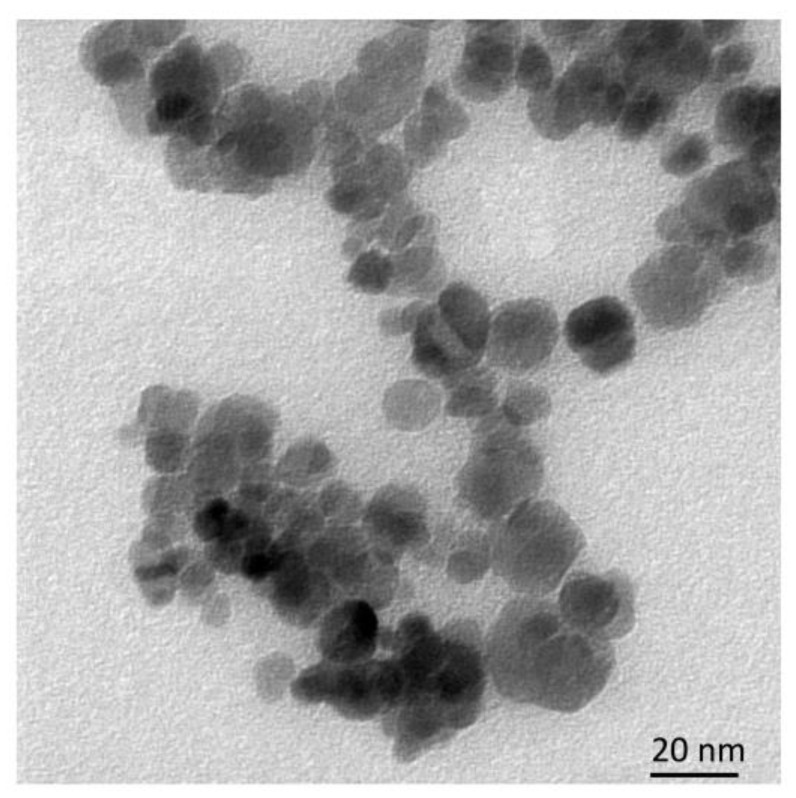
TEM of Fe_3_O_4_-TMSPM-PAlAm/Gd-DTPA-GDD.

According to DLS analysis performed in PBS solution at 36.6 °C, the average diameter of the initial Fe_3_O_4_ NPs is 152 ± 15 nm, while that of the resulting NPs (Fe_3_O_4_-TMSPM-PAlAm/Gd-DTPA-GDD) is 173 ± 19 nm. The average size of NPs obtained by TEM is 25 nm for Fe_3_O_4_-TMSPM-PAlAm/Gd-DTPA-GDD, which is a much smaller value than the measured DLS. This is because DLS determines the hydrodynamic diameter and shows the degree of dispersion of NPs. In a real liquid, under the given conditions, NPs tend to form aggregates of several stuck-together particles. The TEM image is presented in Figure 7.

**Figure 8:**
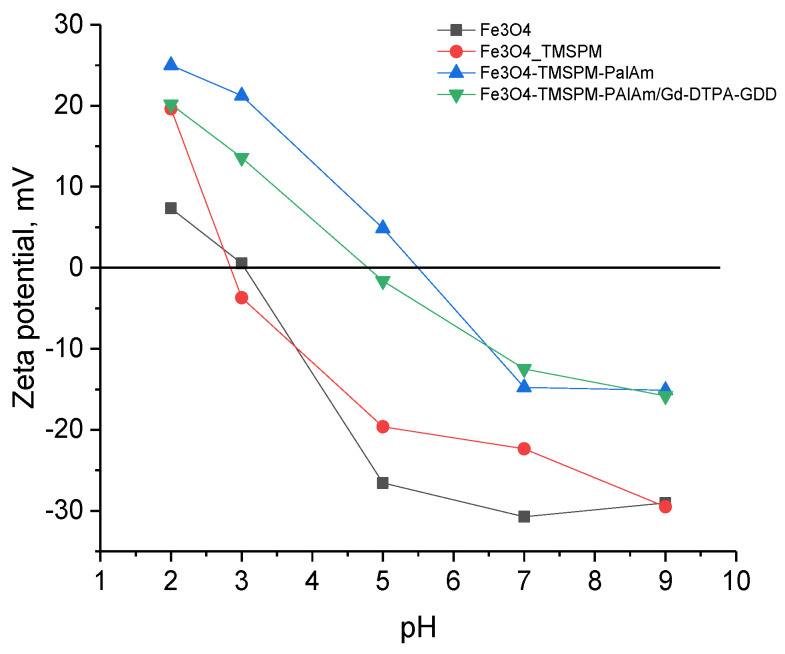
The zeta potential of the obtained nanoparticles.

Data on the zeta potential of the obtained nanoparticles are presented in Figure 8. Zeta potential was measured in ELS mode (using Smoluchowski’s approximations) in PBS solution at different pH values, adjusted using phosphoric acid and sodium hydroxide. Fe_3_O_4_ shows a typical dependence of zeta potential on pH, with an isoelectric point at 3.1. The variation in zeta potential of the initial nanoparticles is due to the presence of OH groups on the Fe_3_O_4_ surface. At pH 2–3, Fe-OH_2_^+^ forms, resulting in a positive zeta potential, while at pH 5–9, Fe-O- forms, resulting in a negative zeta potential. Modification of Fe_3_O_4_ NPs with PAlAm shifts the isoelectric point to the right, up to 5.50, indicating more basic properties of the NPs due to the presence of amino groups on their surface. At pH 2–3, Fe_3_O_4_-TMSPM-PAlAm NPs exhibit NH_3_^+^ groups, resulting in a high zeta potential of 25 mV. Adsorption of Gd-DTPA-GDD slightly shifts the isoelectric point to the left (4.79) due to the presence of carboxyl groups in DTPA. Based on the zeta potential values, Fe_3_O_4_-TMSPM-PAlAm NPs show greater stability in acidic media compared to the initial Fe_3_O_4_ NPs, while at pH 7, the stability decreases as the zeta potential decreases.

**Figure 9:**
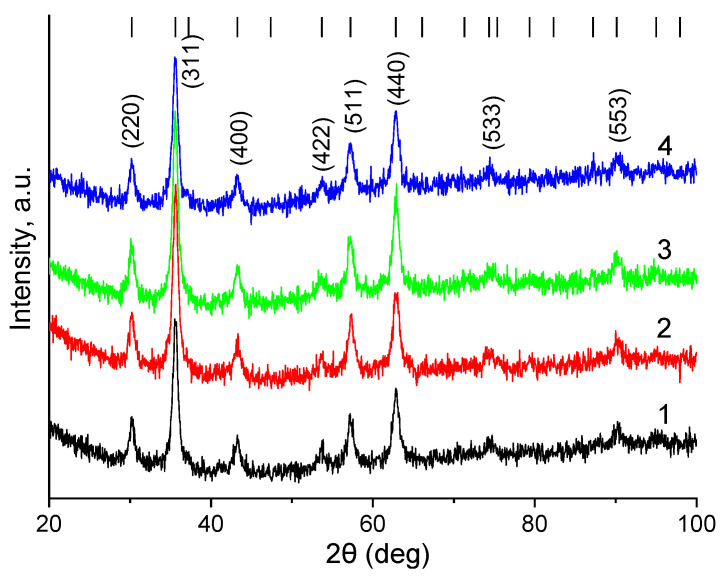
X-ray diffraction patterns of the obtained samples. 1—Fe_3_O_4_, 2—Fe_3_O_4_-TMSPM, 3—Fe_3_O_4_-TMSPM-PAlAm, 4—Fe_3_O_4_-TMSPM-PAlAm/Gd-DTPA-GDD.

The phase composition of the synthesized samples, the crystal lattice parameter, and crystallite size were determined using powder X-ray diffraction. The diffraction patterns for samples are presented in Figure 9.

Crystallography Open Database Fe_3_O_4_ (magnetite) card No. 1011032 with space group Fm-3m (vertical stripes in Figure 8) was used as a reference powder diffraction card. It can be noted that the diffraction patterns contain the most intense reflections characteristic of iron oxides with a spinel crystal structure. These reflections include peaks from the crystallographic planes (220), (311), (511), and (440). This indicates the formation of single-phase magnetite particles using the chosen synthesis method. As can be seen from the diffraction patterns, the reflections have a high full width at half maximum (FWHM), which indicates the small size of the coherent scattering regions. This is caused by the nano size of the resulting magnetite particles and their low crystallinity. Table 4 shows the calculated values of the crystal lattice parameter and crystallite size d: after modification, these parameters change slightly, which indicates the preservation and non-oxidation of magnetite NPs.

**Figure 10:**
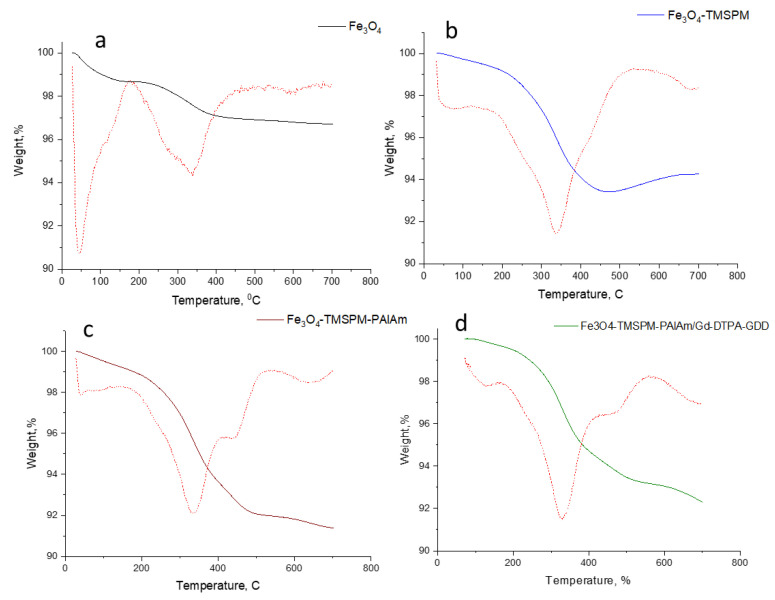
TGA analysis of Fe_3_O_4_ NPs at different stages of modification for Fe_3_O_4_ (**a**), Fe_3_O_4_-TMSPM (**b**), Fe_3_O_4_-TMSPM-PAlAm (**c**), and Fe_3_O_4_-TMSPM-PAlAm/Gd-DTPA-GDD (**d**) samples.

The thermogravimetric analysis (TGA) of F_3_O_4_-NPs at different stages of modification is presented in Figure 10.

Initial Fe_3_O_4_ NPs underwent two mass losses: the first around 100 °C—1.5% corresponding to water removal—and the second at 300 °C—also 1.5% corresponding to the removal of OH groups [48]. The initial Fe_3_O_4_ NPs display high thermal stability, and in the range of up to 700 °C, the weight loss was 3.31%. After modification with TMSPM, there was a more substantial mass drop of 6.7% observed at 340 °C, indicating that 3.4% of the sample consists of polycondensed silane. Further modification through graft polymerization with polyallylamine results in a weight decrease of up to 8.65%, with two peaks of weight loss at 300 °C and 445 °C. Compound immobilization on Fe_3_O_4_ NPs results in minor changes in TGA compared to the Fe_3_O_4_-TMSPM-PAlAm sample.

**Figure 11:**
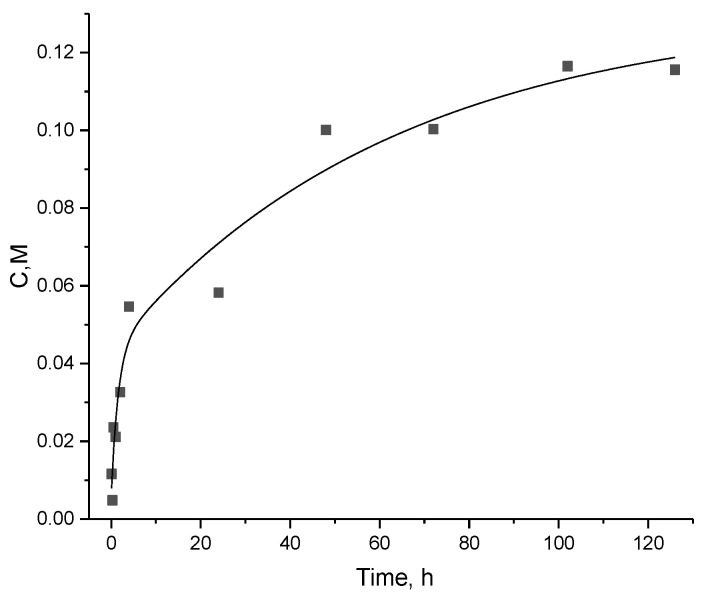
The release of Gd-DTPA-GDD in a phosphate-buffered saline (PBS) solution.

The investigation of Gd-DTPA-GDD release in a phosphate-buffered saline (PBS) solution is illustrated in Figure 11.

The observation extended over a duration of 126 h at a controlled temperature of 36.6 °C. The graphical representation of our observations highlights a notable surge in Gd-DTPA-GDD release within the initial 4 h, suggesting an early and significant release profile. Within the first 4 h of the study, a substantial portion, specifically 61.74% of the total gadolinium content, was released. The subsequent 48 h witnessed a continued release, albeit at a reduced rate, with 30.4% of the total gadolinium released. In the extended observation period of 78 h, a minimal release of 1.7% was recorded. The time-dependent release percentages highlight the dynamic nature of Gd-DTPA-GDD release from modified magnetic NPs. These findings provide information for optimizing materials with controlled release properties in this specific environment.

**Figure 12:**
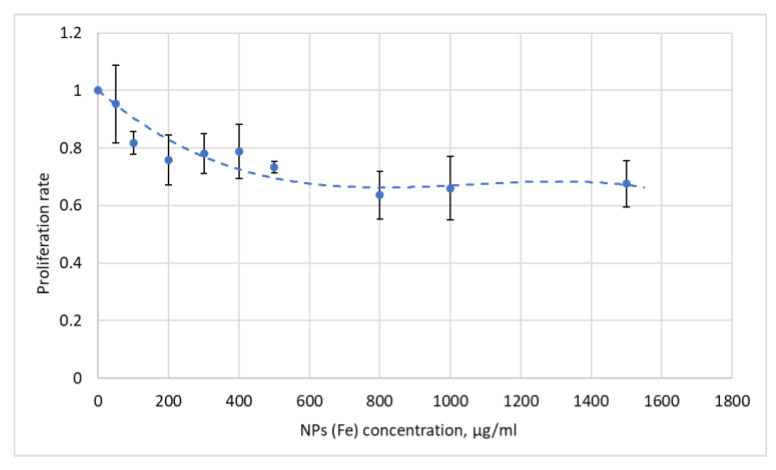
T98G cell proliferation after incubation with NPs.

The cytotoxicity of nanoparticles was evaluated by their effect on cell proliferation. NPs showed tolerable toxicity towards T98 cells along the whole range of concentrations with less toxic concentrations up to 400 µg/mL of Fe (Figure 12), which allows planning further experiments to test NPs on animal tumor models, as therapeutic concentrations in the tumor tissues are declared as 20–30 µg/mL.

**Figure 13:**
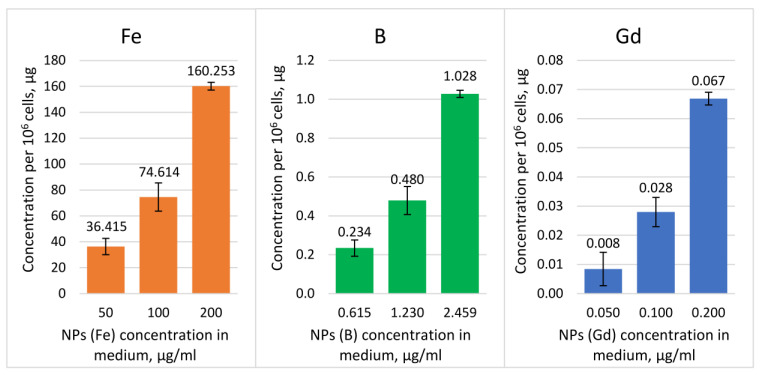
Fe, B, and Gd accumulation per 10^6^ T98G cells after incubation with NPs.

The accumulation in tumor cells represented the possibility of irradiation experiments (Figure 13).

Despite the predominant amount of iron in the nanoparticles, the accumulation of boron and gadolinium indicated the preservation of particle integrity. We calculated the accumulation per million cells since the determination of the concentration of elements per mass may not be accurate due to the small mass of cells and the influence of the mass of the remaining medium in the tube. Then, we calculated the number of atoms of the elements per cell to validate the potential therapeutic efficiency using the following formula:

$$N=\left(\frac{C}{m}\times N_{A}\right)/10^{6},$$

where *N* is the number of atoms per cell, *C* is the amount of the element in 10^6^ cells (g, or μg/10^6^), *m* is the molar mass of the element (g/mol), and *N_A_* is Avogadro’s number (≈6.022 × 10^23^).

The results of the calculations are presented in Table 5.

Knowing that ~10^9^ of ^10^B atoms are necessary for effective boron neutron capture therapy [3,27], we presumed that even with the existing proportions of the elements, the amount of boron is sufficient to trigger the necessary neutron capture and potentially control tumor cell growth. Thus, the boron concentrations of 0.234, 0.480, and 1.028 μg in 10^6^ cells equaled 1.306 × 10^10^, 2.670 × 10^10^, and 5.724 × 10^10^ atoms per tumor cell, which was an order of magnitude higher than the required for BNCT values and indicated the potential efficiency of neutron capture by boron inside tumor cells.

Though the optimal ^157^Gd concentration for successful NCT was considered to be 50–200 μg/g tumor tissues [49], in the case of gadolinium, the number of atoms per tumor cell, as well as its effective concentration, are not universally specified or standardized across studies. Thus, Ho et al. (2020) developed ultra-small gadolinium oxide nanoparticles with cancer-targeting properties, which led to significant U87 tumor size reduction in immunodeficient mice after NCT, and the maximum Gd concentration was measured at between 2 and 2.5 μg/g of tumor tissue [50]. In our study, for gadolinium concentrations of 0.008, 0.028, and 0.067 μg in 10^6^ cells, the number of atoms per cell approximately equaled 3.231 × 10^7^, 1.072 × 10^8^, and 2.561 × 10^8^. Given that Gd-NCT produces gamma rays, which travel further than the alpha particles in BNCT, the required concentration of gadolinium per cell may not need to be as high.

This study has several limitations that need to be addressed. Despite their low toxicity, the NPs contain a much higher percentage of Fe at this stage of development than B or Gd. Due to the much higher concentration of Fe in the NPs, we planned cell experiments based on it. Since B and Gd play a key role in neutron capture therapy, increasing their proportion in the NPs and planning the experiment based on their concentration remains a priority. The existing relatively low content of boron and gadolinium may lead to the use of high amounts of NPs to reach therapeutic concentrations of boron and gadolinium, which will require their better dispersion in water. Further experiments might include incorporating NPs into shells of sugars and polymers, such as hyaluronic acid or hydroxyethyl cellulose, proteins, or other molecules to stabilize NPs in water. Such incorporation in the presence of the Gd-DTPA-GDD compound may influence NP properties and their distribution within the body and interaction with neutron flux.

The process of gadolinium desorption from nanoparticles also remains an issue to be further studied and modified. Given that we do not yet know how long the particles may take to accumulate in the tumor and how they will behave in the bloodstream, the optimal time of gadolinium desorption is still a question for further investigation. Moreover, the desorption could influence gadolinium accumulation in tumor cells to some extent. Though we kept the stock solutions frozen between the experiments, some amount of gadolinium could leave the core particles during melting and incubation with the medium, which could lead to its partial accumulation separately without NPs or lack of Gd accumulation. However, due to different mechanisms of accumulation (endocytosis in the case of NPs and trans-membrane transport in the case of low-molecular compounds), we cannot exactly point out what mechanisms were behind accumulation and to what extent. Moreover, we cannot definitely state that the desorption improved or disrupted gadolinium accumulation in cells, leaving these questions for further studies.

Also, in this study, we used the cell culture most comparable to difficult-to-treat brain tumors. However, this cell line is one of the artificially transplantable well-studied glioma cells. While in real practice we deal with a variety of variations of tumor cells, which may create obstacles to the application of nanoparticles in clinical practice, even if they show high efficacy in the preclinical stage. Therefore, the next stage of our research will be a phase of preclinical experiments using several cell lines and tumor models in laboratory animals, including testing the toxicity of nanoparticles and their accumulation in tumor tissue, followed by neutron irradiation to test the effect of neutron capture therapy.

The tumor-targeting properties of NPs are currently under development, and various approaches can be utilized to deliver our NPs to specific tumors. Among these approaches, we are considering further surface modification using apolipoproteins to target low-density lipoprotein (LDL) receptors, which are abundant on the surfaces of tumor cells [51]. Additionally, the magnetic properties of the NPs allow for active relocation and maintenance of their local concentration within a specific area of a biological system [52].

Regarding elemental composition, we initially used natural elements to synthesize NPs, whereas ^10^B and ^157^Gd isotopes are required for efficient NCT. After refining the NP composition and developing their active tumor-targeting system, natural elements will be replaced with the corresponding isotopes.

## 4. Conclusions

We introduced a novel approach for the modification of Fe_3_O_4_ NPs by incorporating boron and gadolinium for their potential use in cancer diagnosis and NCT. The particles were characterized by EDA, TEM, TGA, XRD, and FTIR spectroscopy. The average size was 25 nm following the final modification, and the content of boron and gadolinium in the carrier was 3.89% and 0.28%, respectively. The maximum (61.74%) desorption of gadolinium occurred within 4 h. The NPs proved to be biocompatible across a broad range of concentrations, enabling their further use in animal experimental tumor models. Increasing the boron and gadolinium content in the NPs and developing an effective tumor-targeting system remain priorities for further studies.

## Figures and Tables

**Table 1:** Elemental content of NPs according to EDA.

Sample	Elemental Content, %						
	Fe	O	C	Si	N	B	Gd
Fe_3_O_4_	63.50 ± 3.78	24.11 ± 4.87	-	-	-	-	-
Fe_3_O_4_-TMSPM	59.61 ± 4.57	28.01 ± 6.16	5.63 ± 1.57	0.98 ± 0.20	-	-	-
Fe_3_O_4_-TMSPM-PAlAm	59.49 ± 5.16	24.08 ± 5.04	6.94 ± 2.05	1.08 ± 0.10	0.7± 0.27	-	-
Fe_3_O_4_-TMSPM-PAlAm/Gd-DTPA-GDD	55.32 ± 4.64	32.21 ± 5.66	6.95 ± 1.31	1.92 ± 0.13	1.12 ± 0.48	3.89 ± 1.18	0.28 ± 0.09

**Table 2:** Dependence of the concentration of amino groups on NPs on the monomer concentration.

Allylamine, M	Concentration of NH_2_-Groups, μM/g
0.48	13.66
0.24	10.30
0.18	15.20
0.12	13.21

**Table 3:** Data on BET surface area, pore volume, and pore width of the samples.

Sample	Specific Surface Area, m^2^/g	Total Pore Volume, cm^3^/g	Average Pore Width, nm	Surface Energy, J/m^2^
Fe_3_O_4_	102.16	0.34	13.31	169.95
Fe_3_O_4_-TMSPM	64.03	0.28	17.29	148.45
Fe_3_O_4_-TMSPM-PalAm	86.95	0.30	13.91	330.32
Fe_3_O_4_-TMSPM-PAlAm/Gd-DTPA-GDD	67.15	0.12	6.97	398.85

**Table 4:** XRD parameters.

No.	a, Å	d, nm
Fe_3_O_4_	8.3608 ± 0.0027	10.64 ± 1.06
Fe_3_O_4_-TMSPM	8.3586 ± 0.0026	9.67 ± 1.43
Fe_3_O_4_-TMSPM-PAlAm	8.3618 ± 0.0025	9.89 ± 1.51
Fe_3_O_4_-TMSPM-PAlAm/Gd-DTPA-GDD	8.3644 ± 0.0029	10.70 ± 0.77

**Table 5:** Elemental content per cell after incubation with NPs.

Element	Amount in 10^6^ Cells, μg	Number of Atoms in a Mol	Number of Atoms per Cell
Fe	36.415	3.925 × 10^17^	3.925 × 10^11^
	74.614	8.043 × 10^17^	8.043 × 10^11^
	160.253	1.728 × 10^18^	1.728 × 10^12^
B	0.234	1.306 × 10^16^	1.306 × 10^10^
	0.480	2.670 × 10^16^	2.670 × 10^10^
	1.028	5.724 × 10^16^	5.724 × 10^10^
Gd	0.008	3.231 × 10^13^	3.231 × 10^7^
	0.028	1.072 × 10^14^	1.072 × 10^8^
	0.067	2.561 × 10^14^	2.561 × 10^8^

## Data Availability

All data generated or analyzed during this study are available upon request from the corresponding authors.
